# Structure-centered portal for child psychiatry research

**DOI:** 10.3389/fninf.2014.00047

**Published:** 2014-04-30

**Authors:** Pallavi Rane, Christian Haselgrove, Steven M. Hodge, Jean A. Frazier, David N. Kennedy

**Affiliations:** Child and Adolescent NeuroDevelopment Initiative, Department of Psychiatry, University of Massachusetts Medical SchoolWorcester, MA, USA

**Keywords:** neurodevelopmental disorders, data integration, knowledge environment, MRI, neuroinformatics

## Abstract

The real world needs of the clinical community require a domain-specific solution to integrate disparate information available from various web-based resources for data, materials, and tools into routine clinical and clinical research setting. We present a child-psychiatry oriented portal as an effort to deliver a knowledge environment wrapper that provides organization and integration of multiple information and data sources. Organized semantically by resource context, the portal groups information sources by context type, and permits the user to interactively “narrow” or “broaden” the scope of the information resources that are available and relevant to the specific context. The overall objective of the portal is to bring information from multiple complex resources into a simple single uniform framework and present it to the user in a single window format.

## Introduction

Neuroimaging studies performed with specific hypothesis in mind are highly informative for learning details about human brain development, elucidating the etiology of numerous psychiatric disorders, and developing ways to remedy them. However, disorder-focused neuroimaging studies have a very precise and narrow objective when it comes to developing a broad understanding the human brain. The data collected during these individual studies can be a resource for extracting additional information about the disorder or the human brain in general. This general concept regarding the latent-content of research data has led to development of numerous neuroimaging data sharing resources, such as NDAR (Hall et al., 2012), NIH Pediatric Database (Evans, 2006), CANDIShare (Kennedy et al., 2012), and ADNI (Jack et al., 2008), which make neuroimaging data available to interested users. In addition to neuroimaging data, hundreds of data and information resources are also available that support dissemination of information related to literature, genetic, derived metadata results, etc. about the brain in health and disease. Despite the burgeoning set of resources hosting research information, attempts to query across these distributed resources are daunting due to variation in the underlying data models, schema and interfaces.

While methods to improve the accessibility of these disparate data resources are underway, an additional consideration needs to be paid to the end user. The Neuroscience Information Framework (NIF) portal (Gardner et al., 2008; Cachat et al., 2012) is an effort to integrate web-based neuroscience resources such as data, materials, and tools. In addition to this general and comprehensive infrastructure, domain-specific solutions are needed in order to meet the real-world needs of the various clinical communities where there is a need to incorporate and integrate these disparate information resources into the routine clinical and clinical research setting.

In this paper we describe the design of a child-psychiatry oriented portal as an effort to deliver a knowledge environment wrapper that provides organization and integration of multiple information sources. Organized semantically by resource context, the portal groups information sources by context type, and permits the user to interactively “narrow” or “broaden” the scope of the information resources that are available and relevant to the specific context. The overall objective of the portal is to bring information from multiple complex resources into a simple single uniform framework and present it to the user in a single interface from which they can easily continue to explore the relevant resources as needed. We will review the conceptual design, describe the methods of implementation, and provide examples of its operation. This will be followed by a discussion of the impact, impediments and future prospects for this type of approach.

## Methods

In this section we review the conceptual design, followed by the practical implementation of the portal. We emphasize the extensible nature of the design, and highlight how content from existing resources is accessed under a common user framework.

### Portal description

The overall system is designed such that a user can generate specific classes of query, identify the various resources that can provide information relevant to the query and then view the results from each of the resources.

**Figure 1:**
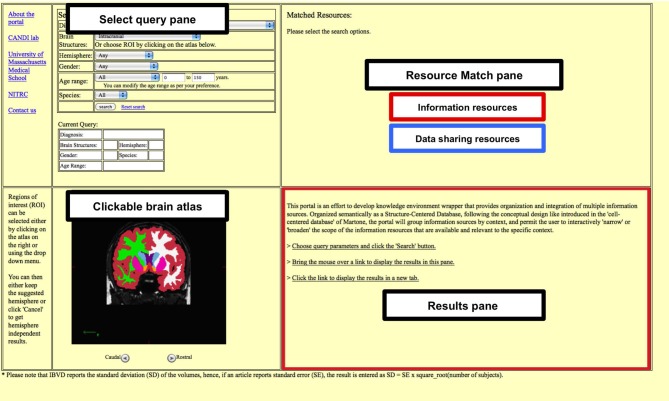
**The four pane window format**. The portal has a four-pane format consisting of Select Query pane, Clickable brain atlas pane, Resource match pane and the Results pane. The Research match pane displays any matching information and data resources. The results for each of the matched resources can either be viewed in the Results pane or be opened in a separate tab.

The portal front end has a four-pane window format (Figure 1): Select Query, Anatomic Atlas, Resource Match and Results. The *Select Query* pane is used to build the desired query. Queries are built out of selection of “contexts,” currently including diagnosis, brain region of interest, gender, age, and species. Diagnoses selection is implemented in a drop down list format. Similarly, brain region of interest can also be chosen from a drop down list or by selecting it in the clickable atlas provided below the search pane. The specific age range can be provided by selecting Young (0–9 years), Adolescent (10–18 years), Young and Adolescent (0–18 years), or Adult (19–150 years). The minimum and the maximum bounds of the age range can be further modified by entering the values directly.

The *Anatomic Atlas* pane supports the user selection of the anatomic context for the *Select Query* pane. This is accomplished through the use of the canvas feature of HTML5. The atlas itself is based on FreeSurfer segmented structural MRI scan of a typically developing 15-year-old female subject. The user can navigate between coronal slices and select regions by mouse click.

The *Resource Match* pane displays links to various available data and information resources, the output for which can be viewed either in the *Results* pane or in a separate web-browser tab. For the data resources a summary of the numbers of datasets available per resource is provided.

### Resources

A specific set of remote resources is currently supported which are queried using the public web services. We make a distinction between two types of resources: information resources and MRI data resources. As will be elaborated upon below, these two classes of resource, and the results returned, require different handling. The following is the set of resources that are currently included:

Information Resources:

*PubMed:* Biomedical literature from MEDLINE, life science journals, and online books).
*Entrez Gene:* Genetic records including nomenclature, reference sequences, maps, pathways, variations, phenotypes, etc.
*IBVD (Kennedy et al., 2003):* Internet Brain Volume Database (IBVD) is a database of volumetric information of different brain structures from over 600 publications and over 15 thousand individual volumes.
*PubBrain (Kalar et al., 2007):* A meta-analysis tool providing numerical and pictorial representation of prevalence of brain structure bibliographic references as identified by the query terms found in PubMed.

MRI Data Resources:

*CANDIShare (Kennedy et al., 2012):* MRI datasets of structural brain images, as well as their anatomic segmentations, demographic, and behavioral data and a set of related morphometric resources for young and adolescent typically developing and psychiatric disorder populations.
*OASIS datasets on XNAT Central:* MRI datasets of very mild to moderate Alzheimer's disease patients including demented and non-demented subjects as well as normal controls between the ages of 18 and 96 years in the cross-sectional dataset, and between the ages of 60 and 96 years in the longitudinal dataset where the subjects are scanned over two or more visits.
^*^OASIS-brains Database (Marcus et al., 2007): OASIS datasets are available through www.oasis-brains.org with additional demographic details such as gender, grouping into demented/non-demented groups and CDR scores (unavailable for download through the XNAT central OASIS release).
*fCON1000 (Biswal et al., 2010):* Neuroimaging database of resting-state functional magnetic resonance imaging data of healthy subjects.
^*^PING (Brown et al., 2012): Large MRI and genetics data set of typically developing children between the ages of 3 and 20 years.
^*^NIH_PD (Evans, 2006): NIH Pediatric database (NIH_PD) of longitudinal MRI data of typically developing children and adolescents scanned during three visits.
^*^ADHD200 (Fair et al., 2012): Publically released dataset of resting-state fMRI and anatomical imaging for 491 typically developing individuals, and 285 in children and adolescents with ADHD between the ages of 7 and 21 years.
^*^ABIDE (Di Martino et al., 2013): Autism Brain Imaging Data Exchange (ABIDE) dataset contains resting state functional imaging and morphometric data from 539 individuals with autism spectrum disorder and 573 typical controls.

Resources marked with ^*^ require some sort of user registration process in order to access the imaging data. While the portal provides simple indication of the types of data that would be obtained with the query (in terms of subjects matching age, gender, and diagnostic characteristics) users are required to acquire their own specific access authentication.

### Operation

Once the user fills in their query terms and clicks the submit button in the “Select Query” pane, resources matching the query are displayed in the Resource Match pane. Each of the information resources can then either be viewed in the Result pane, or opened in a separate tab. For all the imaging databases, the demographic information of available data is displayed in the result pane and the user is directed to the respective websites in order to complete any necessary registration process in order to download the data.

When queries are run against IBVD, CANDIShare, OASIS, fCON1000, PING, NIH_PD, ADHD200, or ABIDE with diagnosis included, the implication is that the user is interested in the contrasts between “typical” and this diagnosis. Therefore, while running the queries on these resources, the query is conducted twice, once for the diagnosis and other context qualifiers, and additionally for age and gender matched normal controls.

Also, data returned from specific resources can be processed locally to derive additional representations of that data. Specific examples of this include automated provision of a z-score table and a z-score plot for the ROI volumes returned from the IBVD results, and a generation of the top five most published genes listing for the Entrez gene results for any given disorder of interest query.

### Implementation

The portal is designed as a stand-alone application. Instead of downloading this application to each user, the application is hosted on a publically available computer and accessed via web-based browser. HTML5 is used to develop the user interface. Dynamic functionality is implemented using JavaScript. The point-and-click brain atlas is implemented using the canvas feature of HTML5. The atlas itself is based on FreeSurfer segmented structural MRI scan of a normal 15-year-old female subject.

**Figure 2:**
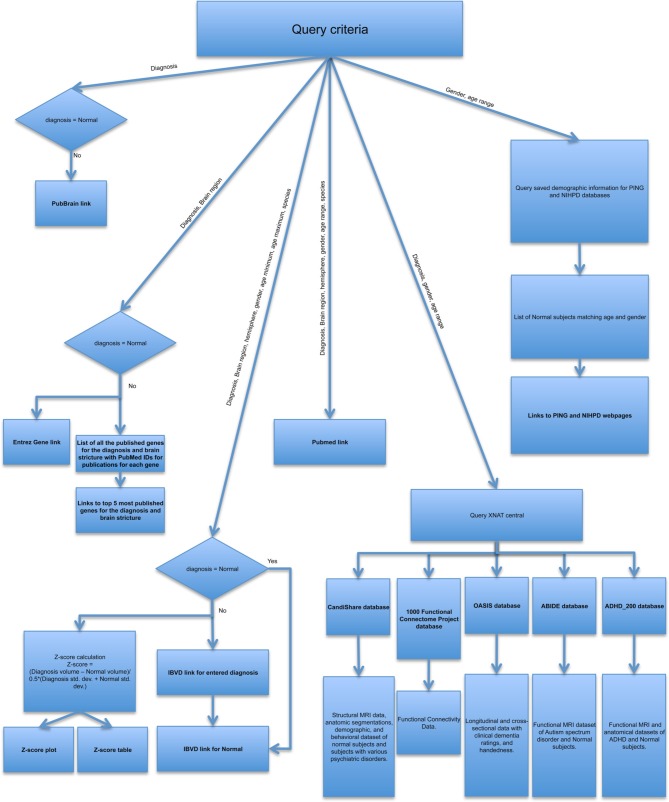
**Flowchart depicting how the query input is tailored to requirements of various resources**.

In the absence of a standard API that facilitates interoperation with all neuroscience resources, we maintain a resource-by-resource catalog of queryable terms and the context that these terms are pertinent to. When queries are implemented we maintain a resource-specific specification of each queryable item and the syntax of the query for that resource. Given the variations between the different resources, the query for each resource is generated independently. Figure 2 provides a pictorial view of how different resources are queried. Either all or a subset of the search criteria is used to generate the query for an individual resource. e.g., PubMed results are based on the diagnosis, brain region, hemisphere, gender, age range as chosen by description (young, adolescent, young, and adolescent, or adult), and species queried, where as PubBrain results are purely based on the diagnosis, gender and age range in years. This approach provides modularity to the portal, making it easier to modify the current queries or add any new resources in future. Another advantage of this approach is that the data is presented in a way that would be most useful to the user. For example, though the IBVD results are limited by age range, we provide a IBVD based z-score plot over the entire age range from young to adult, hence giving the user an overview of changes in volumes of the ROI as a factor of age.

CANDIShare, fCON, ABIDE, ADHD200, and OASIS datasets available through XNAT are queried using Python and pyxnat (Schwartz et al., 2012). NIH pediatric database and PING database are currently not available for direct web query. The results for these resources are made available to the user by querying the demographics available to us. Currently each resource query is custom created (e.g., for IBVD the age range is inputted in the form of minimum and maximum age as opposed to PubMed for which either of young, adolescent or old is used). The results frame utilizes the inline frame feature, hence enabling display of various resource webpages in the same window.

### Local data manipulation

As indicated above, the portal supports a layer of local analyses that can be inserted to process or condition the results of each of the queries to add information or contest. We demonstrate two examples of this local processing that enhance the interpretational value of information returned from the query in support of the clinical end user.

First, the anatomic volume results from IBVD query for a disorder of interest and healthy control groups are represented as a flat table and a graph of raw volumes. A common way of interpreting these results would be for the end user to further parse these results to find matching disorder—normal control results pairs that originate from the same article. These results can then be converted in to a z-score, which is a ratio of difference between the disorder and control group mean to the average standard deviation of the two groups, using the formula stated below. Any articles that might have multiple disorder-normal pairs that can't be separated using gender or hemisphere information are marked as multiple matches. The results are provided to the user as a table as well as a z-score vs. age plot. This sequence of data interpretation steps is automated in the portal in order to provide the end user an added context to the results that are returned. The multiple-matched results are not included in the plot.

$$\text{Z-score}=\frac{\begin{array}{l} (\text{disorder group mean volume}-\\ \;\text{control group mean volume}) \end{array}}{\begin{array}{l} {}[0.5\times (\text{disorder volume std}+\\ \;\text{control volume std})] \end{array}}$$

Finally, a trend-line is generated for the z-score vs. average age plot using the locally weighted scatterplot smoothing (lowess) non-parametric regression (Cleveland, 1979; Cleveland and Devlin, 1988).

As a second example of local result manipulation, we consider the Entrez gene database query result. Initially, this query provides a list of associated genes that is ordered relative to last update of their Entrez gene record (such that the most recently published gene on top of the list). However, as the list of genes returned from a query becomes large, recency of record update is not the optimum criterion for identifying the most salient genetic implications. In this case, the portal will run a process that takes these results and rank order organizes it with respect to the number of publications per gene for the query. The top five of the most published genes are presented under the Gene tab along with a list of all the genes published for that disorder—ROI combination and PubMed IDs of publications for each gene.

**Table:** 

**Resource**	**Diagnosis**	**Brain structure**	**Hemisphere**	**Gender**	**Age range**	**Species**
*PubMed*	Bipolar disorder^a^	Amygdala	–	Female	Young	Human
*EntrezGene*	Bipolar disorder	Amygdala	–	–	–	–
*IBVD*	Bipolar disorder	Amygdala	Left	Female	Age min = 0 Age Max = 9	Human
	Normal	Amygdala	Left	Female	Age min = 0 Age Max = 9	Human
*PubBrain*	Bipolar disorder	–	–	–	–	–
*CANDIShare*	Bipolar disorder	-	-	F^*^	Age min = 0 Age Max = 9	–
	Normal^b^	–	–	F^*^	Age min = 0 Age Max = 9	–
*OASIS datasets on XNAT*	–^**^	–	–	–	Age min = 0 Age Max = 9	–
*OASIS-brains database*	CDR score =”” or CDR score = 0	–	–	F^*^	Age min = 0 Age Max = 9	–
*fCON1000*	Normal	–	–	F^*^	Age min = 0 Age Max = 9	–
*PING*	Normal^#^	–	–	F^*^	Age min = 0 Age Max = 9	–
*NIH_PD*	–^##^	–	–	F^*^	Age min = 0 Age Max = 9	–
*ADHD200*	Control^b^	–	–	F^*^	Age min = 0 Age Max = 9	–
*ABIDE*	Typically developing^b^	–	–	F^*^	Age min = 0 Age Max = 9	–

a Databases such as PubMed expand individual search criterion to match their own terminology.

b Based on a given resource, the search parameters are modified to fit the reported diagnosis, such as “Typically developing” for ABIDE, “Control” for ADHD200, and “Normal” for CANDIShare.

* F for female is compared with the capitalized first letter of the reported gender to determine a match.

** Since diagnosis is not available as a part of OASIS dataset demographics on XNAT, it is not queried.

# PING dataset limits its subjects to those without any confirmed diagnosis of autism, mental retardation, bipolar disorder, schizophrenia, or any neurological disorder such as cerebral palsy, fetal alcohol syndrome, Down's syndrome, fragile X, cerebral neoplasm, bacterial meningitis, epilepsy, and hence the subjects are considered “Typically developing.”

## NIH_PD exclusion criteria included diagnosis for any major medical illness, congenital abnormalities, heart problems, cancer, lead poisoning, seizures, CNS Infection, head injury, significant hearing loss, language disorder, mood disorder, Conduct, AD/HD, Tic, Eating disorders, as well as, presence of bipolar disorder, chronic depression, psychotic, AD/HD, drug dependence, or PDD in first degree relatives. Hence, can be considered typically developing and no diagnosis is reported.

### Use cases

We illustrate the query building functionality through examination of the details for a sample query:

Disorder: Bipolar Disorder
Brain Structure: Amygdala
Hemisphere: Left
Gender: Female
Age: Young or 0–9 years
Species: Human

For each resource, the following table shows the mapping of the context terms to the actual query.

### User access

The portal is freely accessible as a website hosted at http://childportal.virtualbrain.org. This host is an Amazon EC2 NITRC-Computational Environment Ubuntu 12.04 platform. The underlying computational power of the EC2 instance can be scaled to meet variations in portal demand.

## Results

**Figure 3:**
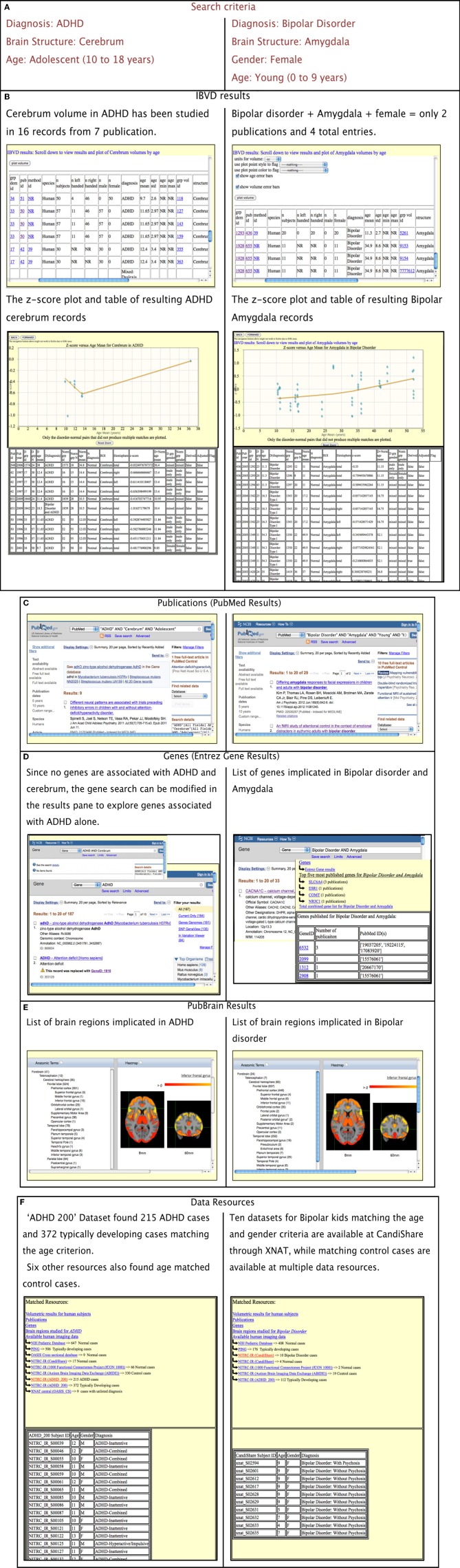
**Portal output based on two separate search criteria. (A)** Search criteria, **(B)** Results of IBVD for each of the queries displayed in tabular as well as z-score plot form, **(C)** Publication results for the queries, **(D)** Entrez Gene result along with the top five most published genes for the disorder and brain region in query, **(E)** PubBrain results for the disorder queried which enlist the brain regions published for that disorder, **(F)** Data resources which can provide the user with MRI data available for the disorder queried as well as normal control data which fits the rest of the query criteria.

The operation of the portal is best illustrated through a number of examples/case studies. Figure 3 provides an overview and comparison of the results of two different contextual queries. The left hand column displays the results for a query on “Diagnosis: ADHD; Brain Structure: cerebrum; and Age: Adolescent.” The right column displays query for “Diagnosis: Bipolar Disorder; Brain Structure: Amygdala; Gender: female; and Age: Young” (Figure 3A). The links are generated for each resource and presented to the user. The specific, query-dependent version for each resource is displayed. As shown in Figure 3B, the IBVD results for the query are presented along with z-score table and plot for the disorder-ROI combination. Similarly, the Entrez Gene results are further processed and presented with the top five most published genes for the disorder-ROI combination, as shown in Figure 3D for the Bipolar-Amygdala query which has only four genes that actually have common publications for Bipolar disorder and Amygdala despite the list of 33 genes produced by Entrez Gene. The user can further manipulate the output for each of the information resource, if necessary. For example, Entrez gene did not have any entries for the combined query of ADHD and cerebrum (Figure 3D). The user can in such cases modify the search in the results pane for that particular resource only to look at gene entries for ADHD alone.

For the MRI data resources, the available data for the disorder as well as normal controls are displayed for the age range in question. If any resource has not specified any disorder in their demographics, those results are displayed as well and listed as “unspecified disorder.” Since many data resources require a user to register with them before the data can be released, the portal points them to the resource websites in case they want to access the data.

## Discussion

Despite the presence of numerous neuroinformatics resources that are available to the clinician, we believe that the Child Psychiatry Portal is the first effort to create a platform to consolidate these data and information resources specifically for the needs of the pediatric psychiatry researcher. Currently the target resources include IBVD, PubMed, Entrez gene, and PubBrain, NIH pediatric database, PING, CANDIShare, 1000 Functional Connectomes Project (FCON), and OASIS longitudinal and cross-sectional studies. We list data that is available through the five resources we query whether it matches the entire query as specified, including diagnosis, or it matches the age range and gender characteristics specified for control (typically developing) subject data. The power of this approach comes not through the complexity of any one query, but rather the collection and integration of a wide variety of resource queries under one application where most of the operational details and idiosyncrasies of the of the individual resources can be initially abstracted away from the end user. Ultimately, use of these varied resources by users not intimately trained in the details of each site will be critical to wide-spread utilization of these many valued data sources.

### Advantages

The first and foremost advantage of this portal is that it brings information from multiple complex resources into a simple single uniform framework without requiring myriad of resource-specific syntax knowledge. Additionally, the portal has an extensible modular architecture. Hence, it can be expanded to include results from any additional resources as and when they become available. We understand that a user might already be aware of some of the individual resources and be well versed in navigating these resources. However, the ability to modify the search parameters for multiple resources simultaneously should be an advantage to the user over having to go through each of the resources individually. This represents a dramatic saving in terms of time in order to look for availability of information from multiple resources every time one needs to modify a search parameter. See the Supplementary Material for the description of performing query on individual resources presented in the portal without the portal.

A second critical advantage of the system is to introduce a data manipulation layer between the raw results from the various resources and the presentation of this information in a form that is best suited to the end user. Databases that do a good job of collecting data cannot anticipate and support every re-use and re-interpretation that can be envisioned for their data. As users develop convenient ways to interpret data, there need to be equally convenient ways to implement and disseminate these views to the end users that are more flexible than building upon the database infrastructure itself.

Finally, within the neuroimaging search functionality, acknowledging that multiple data sources (implemented using multiple data hosting platforms) will always exist and that the content from these sources will ultimately need to be pooled, requires the development of a “higher-order” search platform that can span a dynamically changing landscape of image data resources. The ability to both quickly and efficiently integrate data sets between sources and discover the presence of additional data sources will grow in importance as the amount of shared image data, number of providers, and variety of access terms increases.

### Limitations

As it can be seen from the results, though numerous studies are published every year, a very small portion of the data is made available and it is further limited in case of studies of psychiatric disorders in children. This highlights the need to promote data sharing to researchers. Currently only a limited number of MRI Data resources are available for downloading patient related imaging data. Despite this, the user can at least take advantage of any available control data, perhaps for integration with their own patient datasets.

Another important thing to note is, not all available data resources follow similar rules for nomenclature. For example, the 1000 functional connectomes (fCON1000) project and the OASIS longitudinal and cross-sectional datasets do not make it explicitly clear in their demographics the diagnosis of their subjects. This information needs to be inferred based on the description provided on their respective webpages as normal controls for the fCON1000, and as probable Alzheimer's Disease if a CDR scores >0 or otherwise healthy controls for the OASIS datasets. Similarly, the OASIS datasets, which actually are available for download through XNAT central, do not provide the gender of the subjects on XNAT central, hence in case of gender specific query, the XNAT central resource gets ignored. As of this writing, PING and NIH pediatric database are not yet available for direct query over the web. In these cases, we have separate access to the demographic information saved locally upon which those specific queries are run. CANDIShare, fCON, and OASIS databases are available through XNAT (NITRC-IR and XNAT central) and have some similarities between their demographics data structure. However, in general we had to run individualized queries for most of the databases, making it an *ad-hoc* peer-to-peer style process as described earlier. We hope that in future there would be developments toward streamlining and homogenizing the way the information in stared and presented. The INCF Neuroimaging Data Sharing task force (Poline et al., 2012) is working on an API which would standardize description of neuroimaging/meta data to facilitate the communication between databases. However, we are still far away from standardization of available research resources, hence necessitating a portal presented in this paper.

## Future work

We will continue expanding the list of available resources as and when they become available and open to be queried. fMRI activation results from the BrainMap (Laird et al., 2005) and SuMSDB (Van Essen et al., 2004) databases are an obvious extension. In addition, bridging between resources that integrate across species will be critical. Adding homology mapping and additional resources like the Allen Brain Institute mouse gene expression database and the CoCoMac database of connectivity will broaden the types of inference that can be supported by the portal environment. We plan to further customize our currently reported results, similar to the brain volume z-score plots or the most published genes, to improve the end usability.

When searching for neuroimaging data using the portal, the user quickly runs into the barriers of publically vs. privately shared data sources. While the portal helps to identify the magnitude of query results that will be found if one has access to these private data sources, users themselves must conform to the various data sharing policies needed for each. Future extensions to the portal that help a user manage their multiple different resource access permissions and facilitate data integration across these multiple sites will be pursued.

In the near future we plan to add a feature to highlight the queried aberrance in the Z-plots. In this fashion, it will become clearer where there is or isn't data available and how that age-range-specific data fits in the context of data from other ages.

The portal currently takes into consideration one disorder and one brain region of interest. In future, we plan to add additional number of disorders to address co-morbidities. We also plan to expand the query to include more than one ROI, so that any commonalities in the results that might exist between multiple brain regions, which could shed more light on the etiology of a disorder, can be made available to the user.

## Conclusion

Despite of these limitations, our portal provides an initial prototype for a homogenized front end for a variety of resources that would ease the burden of information integration for child-psychiatry researchers.

### Conflict of interest statement

The authors declare that the research was conducted in the absence of any commercial or financial relationships that could be construed as a potential conflict of interest.

## References

[B1] Biswal B. B. Mennes M. Zuo X. N. Gohel S. Kelly C. Smith S. M. (2010). Toward discovery science of human brain function. Proc. Natl. Acad. Sci. U.S.A. 107, 4734–4739 10.1073/pnas.0911855107 20176931 PMC2842060

[B2] Brown T. T. Kuperman J. M. Chung Y. Erhart M. Mccabe C. Hagler D. J. (2012). Neuroanatomical assessment of biological maturity. Curr. Biol. 22, 1693–1698 10.1016/j.cub.2012.07.002 22902750 PMC3461087

[B3] Cachat J. Bandrowski A. Grethe J. S. Gupta A. Astakhov V. Imam F. (2012). A survey of the neuroscience resource landscape: perspectives from the neuroscience information framework. Int. Rev. Neurobiol. 103, 39–68 10.1016/B978-0-12-388408-4.00003-4 23195120

[B4] Cleveland W. S. (1979). Robust locally weighted regression and smoothing scatterplots. J. Am. Stat. Assoc. 74, 829–836

[B5] Cleveland W. S. Devlin S. J. (1988). Locally weighted regression: an approach to regression analysis by local fitting. J. Am. Stat. Assoc. 83, 596–610

[B6] Di Martino A. Yan C. G. Li Q. Denio E. Castellanos F. X. Alaerts K. (2013). The autism brain imaging data exchange: towards a large-scale evaluation of the intrinsic brain architecture in autism. Mol. Psychiatry 18:78 10.1038/mp.2013.78 23774715 PMC4162310

[B7] Evans A. C. (2006). The NIH MRI study of normal brain development. Neuroimage 30, 184–202 10.1016/j.neuroimage.2005.09.068 16376577

[B8] Fair D. A. Nigg J. T. Iyer S. Bathula D. Mills K. L. Dosenbach N. U. (2012). Distinct neural signatures detected for ADHD subtypes after controlling for micro-movements in resting state functional connectivity MRI data. Front. Syst. Neurosci. 6:80 10.3389/fnsys.2012.00080 23382713 PMC3563110

[B9] Gardner D. Akil H. Ascoli G. A. Bowden D. M. Bug W. Donohue D. E. (2008). The neuroscience information framework: a data and knowledge environment for neuroscience. Neuroinformatics 6, 149–160 10.1007/s12021-008-9024-z 18946742 PMC2661130

[B10] Hall D. Huerta M. F. McAuliffe M. J. Farber G. K. (2012). Sharing heterogeneous data: the national database for autism research. Neuroinformatics 10, 331–339 10.1007/s12021-12012-19151-12024 22622767 PMC4219200

[B11] Jack C. R. Jr. Bernstein M. A. Fox N. C. Thompson P. Alexander G. Harvey D. (2008). The Alzheimer's disease neuroimaging initiative (ADNI): MRI methods. J. Magn. Reson. Imaging 27, 685–691 10.1002/jmri.21049 18302232 PMC2544629

[B12] Kalar D. Poldrack R. Parker D. S. Torvik V. Smalheiser N. Bilder R. M. (2007). PubBrain: an interactive website for literature visualization and exploration. Organ. Hum. Brain Mapp. Abstr.

[B13] Kennedy D. N. Haselgrove C. Hodge S. M. Rane P. S. Makris N. Frazier J. A. (2012). CANDIShare: a resource for pediatric neuroimaging data. Neuroinformatics 10, 319–322 10.1007/s12021-12011-19133-y 22006352 PMC3417225

[B14] Kennedy D. N. Haselgrove C. McInerney S. (2003). MRI−based morphometric analysis of typical and atypical brain development. Ment. Retard. Dev. Disabil. Res. Rev. 9, 155–160 10.1002/mrdd.10075 12953294

[B15] Laird A. R. Lancaster J. J. Fox P. T. (2005). Brainmap. Neuroinformatics 3, 65–77 10.1385/NI:3:1:065 15897617

[B16] Marcus D. S. Wang T. H. Parker J. Csernansky J. G. Morris J. C. Buckner R. L. (2007). Open access series of imaging studies (OASIS): cross-sectional MRI data in young, middle aged, nondemented, and demented older adults. J. Cogn. Neurosci. 19, 1498–1507 10.1162/jocn.2007.19.9.1498 17714011

[B17] Poline J. B. Breeze J. L. Ghosh S. Gorgolewski K. Halchenko Y. O. Hanke M. (2012). Data sharing in neuroimaging research. Front. Neuroinform. 6:9 10.3389/fninf.2012.00009 22493576 PMC3319918

[B18] Schwartz Y. Barbot A. Thyreau B. Frouin V. Varoquaux G. Siram A. (2012). PyXNAT: XNAT in Python. Front. Neuroinform. 6:12 10.3389/fninf.2012.00012 22654752 PMC3354345

[B19] Van Essen D. Dickson J. Harwell J. Hanlon D. W. (2004). SumsDB: online access to surface-based representations of cerebral and cerebellar cortex in primates and rodents, in Human Brain Project Annual Meeting (Bethesda, MD).

