# Supplementary material for: Structure-centered portal for child psychiatry research
Source: Front Neuroinform. 2014 Apr 30;8:47. doi: 10.3389/fninf.2014.00047 (PMC4012203; doi:10.3389/fninf.2014.00047)
Supplement: Supplementary file 1 [file DataSheet1.PDF]

**Supplementary information**

**Performing the query for individual resources presented in the portal**

For the query: ADHD + Cerebrum + Adolescent

*IBVD*

1. Look up IBVD url (http://www.cma.mgh.harvard.edu/ibvd/)
2. Go to site
3. Click on 'search'
4. Click structure, cerebrum
5. Click diagnosis 'normal' and shift click diagnosis 'ADHD' and select 'is exactly'
6. Select species 'human'
7. For hemisphere, unclick 'any', 'right', 'left', 'NR'
8. Enter 10 and 18 for age min & max
9. Hit submit

*To generate Z-plot*

1. Run the query for ADHD and Cerebrum in IBVD using previous steps but for entire age range
2. Download results to table
3. Re-run the query for Cerebrum in IBVD for 'Normal' as diagnosis
4. Download results to table
5. Import into excel
6. Find records that are diagnostic comparison matches from each paper using publication ID, gender, hemisphere as matching parameters
7. Calculate their Z-scores

8. Plot

*PubMed:*

1. Start new browser tab/window
2. Go to PubMed url
3. Enter query

*Entrez Gene*

1. Start new browser tab/window
2. Go to website http://www.ncbi.nlm.nih.gov/pubmed
3. Enter query
4. See that there is no results
5. Remove adolescent qualifier
6. See that there are no results
7. Query only on ADHD
8. Go throughout the 222 gene results
9. Compile number of publications indicated on the gene page
10. Sort by number of publications
11. If the Query for disorder + adolescent did infact produce gene results on Entrez gene, e.g. Bipolar Disorder and Amygdala which produces 33 genes as result, the use will have to go through 33 PubMed searches for Gene Name and Bipolar disorder in order to figure out that the actual number of Genes that are published for Bipolar disorder are infact much more limited as depicted in Figure 3

*PubBrain*

1. Start new browser tab/window

2. Go to PubBrain url (http://www.pubbrain.org/)
3. Enter query
4. Discover PubBrain bug when multiple search terms are entered
5. Kill browser page and redo query with just ADHD

*NIH Pediatric Database*

1. Start new browser tab/window
2. Find the correct url
3. Go to website
4. Login and authenticate
5. Find their search page
6. Search for ADHD data in the 10-18 year old range
7. Search for typically developing subjects (or whatever they're called in this database) in the 10-18 year old range

*PING, ADHD-200, ABIDE, fCON 1000, CANDIShare (all are accessible out of NITRC-IR)*

1. Start new browser tab/window
2. Find NITRC url
3. Go to NITRC site
4. Go to Image Repository
5. Authenticate to NITRC
6. Make sure you are a member (or request access) to the PING, and fCON 1000 projects (for access to fCON 1000, ADHD-200 and ABIDE data sets)
7. Go to the advanced search

8. Pivot on 'MR Sessions'
9. Select detailed data type for 'MR Sessions'
10. Enter age range in MR Sessions tab
11. Capture this result and download
12. Sort on 'Group'
13. Select subset of data with Group = ADHD as patient population
14. Select subset of data with Group = 'Normal' OR 'Typically Developing' OR 'Control' as control group

*XNAT Central*

1. Start new browser tab/window
2. Find url
3. Go to website
4. Go to MR search tab
5. Select age range 10-18
6. Select project 'Central OASIS_CS'
7. Submit query

***At this point, if one decides to include the younger population to the search by making the age range searched from 'Adolescent' to 'Young and adolescent' because of some new information he/she has come across, the entire process will have to be repeated with the new age range of say 0 – 18 years.***
